# Exercise Intolerance Is Associated with Cardiovascular Dysfunction in Long COVID-19 Syndrome

**DOI:** 10.3390/jcm13144144

**Published:** 2024-07-16

**Authors:** Angelos Vontetsianos, Nikolaos Chynkiamis, Maria Ioanna Gounaridi, Christina Anagnostopoulou, Christiana Lekka, Stavroula Zaneli, Nektarios Anagnostopoulos, Evangelos Oikonomou, Manolis Vavuranakis, Nikoletta Rovina, Andriana I. Papaioannou, Georgios Kaltsakas, Nikolaos Koulouris, Ioannis Vogiatzis

**Affiliations:** 1 Rehabilitation Unit, 1st Respiratory Medicine Department, “Sotiria” Hospital, National and Kapodistrian University of Athens, 11527 Athens, Greece; agelvonte@gmail.com (A.V.); christinaanagnosto@gmail.com (C.A.); christiana.lekka@gmail.com (C.L.); stavzaneli@gmail.com (S.Z.); aris.anag@yahoo.gr (N.A.); papaioannouandriana@gmail.com (A.I.P.); georgios.kaltsakas@gstt.nhs.uk (G.K.); koulnik@med.uoa.gr (N.K.); ioannis.vogiatzis@northumbria.ac.uk (I.V.); 2 Thorax Research Foundation, 11521 Athens, Greece; 3 3rd Department of Cardiology, Sotiria Chest Disease Hospital, Medical School, National and Kapodistrian University of Athens, 11527 Athens, Greece; mar.gounaridi@gmail.com (M.I.G.); boikono@gmail.com (E.O.); vavouranakis@gmail.com (M.V.); 4 1st Respiratory Medicine Department, “Sotiria” Hospital, National and Kapodistrian University of Athens, 11527 Athens, Greece; nikrovina@med.uoa.gr; 5 Lane Fox Respiratory Service, Guy’s and St Thomas’ NHS Foundation Trust, London SE1 7EH, UK; 6 Centre of Human and Applied Physiological Sciences, Faculty of Life Sciences and Medicine, King’s College London, London SE1 1UL, UK; 7 Department of Sport, Exercise and Rehabilitation, Faculty of Health and Life Sciences, Northumbria University Newcastle, Newcastle upon Tyne NE1 8ST, UK

**Keywords:** long COVID-19 syndrome, cardiopulmonary exercise test, echocardiography, exercise tolerance

## Abstract

**Background/Objectives:** Cardiorespiratory complications are commonly reported among patients with long COVID-19 syndrome. However, their effects on exercise capacity remain inconclusive. We investigated the impact of long COVID-19 on exercise tolerance combining cardiopulmonary exercise testing (CPET) with resting echocardiographic data. **Methods:** Forty-two patients (55 ± 13 years), 149 ± 92 days post-hospital discharge, and ten healthy age-matched participants underwent resting echocardiography and an incremental CPET to the limit of tolerance. Left ventricular global longitudinal strain (LV-GLS) and the left ventricular ejection fraction (LVEF) were calculated to assess left ventricular systolic function. The E/e’ ratio was estimated as a surrogate of left ventricular end-diastolic filling pressures. Tricuspid annular systolic velocity (SRV) was used to assess right ventricular systolic performance. Through tricuspid regurgitation velocity and inferior vena cava diameter, end-respiratory variations in systolic pulmonary artery pressure (PASP) were estimated. Peak work rate (WRpeak) and peak oxygen uptake (VO_2_peak) were measured via a ramp incremental symptom-limited CPET. **Results:** Compared to healthy participants, patients had a significantly (*p* < 0.05) lower LVEF (59 ± 4% versus 49 ± 5%) and greater left ventricular end-diastolic diameter (48 ± 2 versus 54 ± 5 cm). In patients, there was a significant association of E/e’ with WRpeak (r = −0.325) and VO_2_peak (r = −0.341). SRV was significantly associated with WRpeak (r = 0.432) and VO_2_peak (r = 0.556). LV-GLS and PASP were significantly correlated with VO_2_peak (r = −0.358 and r = −0.345, respectively). **Conclusions:** In patients with long COVID-19 syndrome, exercise intolerance is associated with left ventricular diastolic performance, left ventricular end-diastolic pressure, PASP and SRV. These findings highlight the interrelationship of exercise intolerance with left and right ventricular performance in long COVID-19 syndrome.

## 1. Introduction

The COVID-19 pandemic has affected more than 650 million people [1], imposing a major health and socioeconomic burden on healthcare systems globally. Even though most patients survive the acute phase of the disease [2], a significant number of them present with persistent symptoms for more than three months after hospital discharge—a condition known as long COVID-19 syndrome. The most commonly reported symptoms include fatigue, dyspnoea and exercise intolerance [3].

A recently published systematic review and meta-analysis reported that exercise capacity, assessed via cardiopulmonary exercise testing (CPET), was reduced in a significant number of patients with long COVID-19 [4]. The potential cardiovascular mechanisms associated with reduced exercise tolerance included chronotropic incompetence and a reduced stroke volume [4].

During the acute phase of COVID-19, the cardiovascular system is affected through multiple mechanisms involving microcirculation injury, endothelial dysfunction, thrombosis activation, inflammation and immune activation [5]. During the long COVID-19 phase, several cardiovascular manifestations have been described to persist for up to one year following convalescence from acute COVID-19. Among other features, cardiovascular involvement in the long phase of the disease may be present with autonomic dysregulation, related to orthostatic hypotension and high or irregular heart rhythms, endothelial dysfunction, impaired left ventricular function, ventriculoarterial uncoupling and exercise intolerance [6,7,8].

Based on such evidence, it was hypothesised that cardiovascular dysfunction could be a potential contributing mechanism to exercise intolerance in patients with long COVID-19 syndrome [9]. The aim of our study was to investigate the association between right and left ventricular performance and exercise tolerance in patients with long COVID-19 syndrome by combining the diagnostic results of cardiopulmonary exercise testing with resting echocardiography.

## 2. Materials and Methods

### 2.1. Study Design

**Figure 1:**
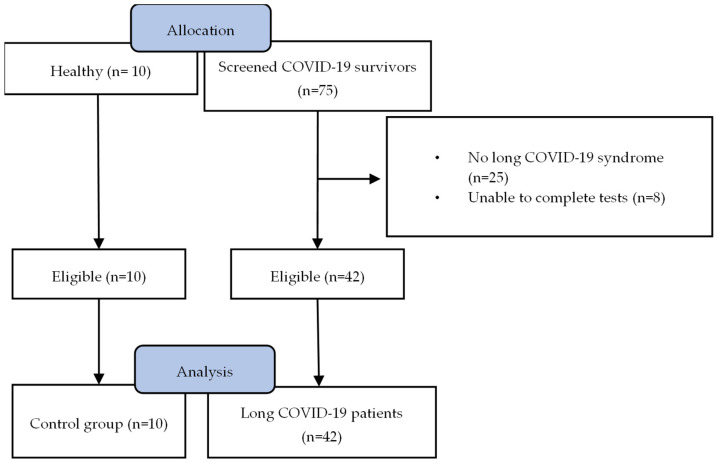
Study flowchart. Following the initial screening, 42 patients and 10 healthy age-matched individuals underwent cardiopulmonary exercise testing (CPET) and resting echocardiography. All participants were included in the final analysis.

This was an exploratory, single-centre, cross-sectional study, investigating the association between cardiac function and exercise intolerance in patients with long COVID-19 syndrome. This study took place at Sotiria General Hospital for Chest Diseases, Athens, Greece, from August 2022 to December 2022. All patients were diagnosed with long COVID-19 based on the persistence of fatigue for a minimum of three months following hospital discharge due to SARS-CoV-2 infection [9,10]. Healthy participants that matched with patients with long COVID-19 for age and BMI served as controls. This study’s flowchart is presented in Figure 1. Exclusion criteria included participation in another clinical trial, history of myocardial infarction, hospitalisation for unstable angina, stroke, coronary artery bypass graft, implantation of a cardiac resynchronisation therapy device, active treatment for cancer, advanced heart failure and heart failure decongestion, acute psychosis, major psychiatric disorder, neurological disease, musculoskeletal problems or substance abuse within 3 months prior to informed consent. Following long COVID-19 diagnosis, informed written consent was obtained from all study participants and a ramp incremental CPET and resting echocardiography test were performed on the same day. The investigations were carried out according to the rules of the Declaration of Helsinki of 1975, and this study was approved by the Sotiria Hospital Ethics Committee (Protocol ID-24633). The present investigation comprises a sub-study of a research project assessing the effects of rehabilitation on CPET outcomes in patients with long COVID-19 syndrome, registered at ClinicalTrials.gov with identifier number NCT05736939.

### 2.2. Respiratory Function Assessment

Standard spirometry was performed with a metabolic cart (Vmax Encore 22: Sensor Medics, Yorba Linda, CA, USA) using the “fast inspiratory manoeuvre” [11]. Static lung volumes were determined by the multiple nitrogen washout technique (Vmax Encore 22 apparatus) [12]. The Diffusing Capacity Of The Lungs For Carbon Monoxide (DLCO) was measured via the single-breath technique (Vmax Encore 22 apparatus) [13]. Predicted values for spirometry, static lung volumes and the DLco were in accordance with the European Community for Coal and Steel [14].

### 2.3. Chronic Dyspnoea and Fatigue Assessment

The assessment of chronic dyspnoea was performed using the modified Medical Research Council Dyspnoea Scale (Mmrc), scale 0–4. Chronic fatigue was assessed via the FACIT questionnaire [15].

### 2.4. Ramp Incremental Cardiopulmonary Exercise Test

Participants performed a ramp incremental CPET to the limit of tolerance on an electromagnetically braked cycle ergometer (Vyaire Medical GmbH, Hoechberg, Germany) with breath-by-breath gas exchange measurements. The exercise protocol was divided into three phases as follows: (a) 3 min of resting baseline measurements, (b) 3 min warm-up of unloaded pedalling and (c) ramp incremental exercise where the work rate was increased by 10–25 Watt every minute to the limit of tolerance. The phase of the incremental exercise did not exceed 10–12 min. The pedalling frequency was set at 50–60 revolutions/min.

Throughout the tests, patients breathed through a pneumotachograph, which was connected to a metabolic cart (Vyntus ONE, Vyaire Medical GmbH, Hoechberg, Germany) for recording the physiological parameters of breathing on a breath-by-breath basis. During each test, arterial oxygen saturation (SpO_2_%) was recorded by an ear pulse oximeter connected to the metabolic cart (Vyntus ONE, Vyaire Medical GmbH, Hoechberg, Germany). Cardiac function was assessed via a 12-lead ECG (Marquette Max, Marquette Hellige GmbH, Freiburg Im Breisgau, Germany), and blood pressure was monitored via an automatic cuff connected to the metabolic cart at predetermined time points. The modified Borg 1–10 scale [16] was used to rate the magnitude of perceived dyspnoea and leg discomfort every 3 min throughout the test and at the end of the exercise test.

### 2.5. Resting Echocardiography

Transthoracic echocardiography tests were conducted with all participants in the left lateral decubitus position, using the Philips EPIQ CVx system (Philips, Andover, MA, USA) and performed by the same expert operator. All Echo studies were analysed using the Philips Q station 3.9 ultrasound software. The Echo protocol included all standard views and measurements according to the American Society of Echocardiography and the European Society of Cardiovascular Imaging [17]. Left ventricular systolic function was evaluated by calculating the left ventricular ejection fraction (LVEF) according to Simpson’s Biplane Method using 4-chamber and 2-chamber views [17]. Concurrently, an automated 2D Cardiac QuantificationA.I system was used. The estimation of the left ventricular global longitudinal strain (LV-GLS) was performed with the Philips Automated Cardiac Motion QuantificationA.I system (aCQM), using the apical 4-, 3- and 2-chamber views, and subsequent to manual adjustments, a deformation assessment through a 2D speckle tracking technology was achieved. Left ventricular end-diastolic filling pressures were estimated through the ratio of early trans-mitral flow velocity to early mitral annular velocity (E/e’) [18]. Right ventricular systolic performance was evaluated using tissue Doppler imaging by calculating the peak tricuspid annular systolic velocity (SRV). End-respiratory variation systolic pulmonary artery pressure (PASP) was calculated with the simplified Bernoulli equation using the maximum tricuspid regurgitation velocity and inferior vena cava diameter end-respiratory variation [19].

### 2.6. Statistical Analysis

Categorical variables were presented as absolute values. Continuous variables were tested for normality with the Kolmogorov–Smirnov test and by plotting p-p plots and visual inspection. Accordingly, normally distributed continuous variables were presented as the mean ± SD. For continuous variables, comparisons between the two studied groups were performed using an independent sample *t*-test. For categorical variables, differences were tested with the chi square test. Correlation analysis was performed using the Pearson correlation coefficient. All calculations were based on two-sided tests. A *p*-value < 0.05 was considered significant. Statistical analysis was performed using IBM SPSS 22 statistical software.

## 3. Results

### 3.1. Study Population

The demographic characteristics of the patients and healthy controls are presented in Table 1. Patients with long COVID-19 had normal lung function and increased levels of chronic fatigue and dyspnoea (Table 1).

### 3.2. Exercise Tolerance

Data from CPET are presented in Table 2. Patients with long COVID-19 syndrome presented with lower exercise tolerance compared to healthy age-matched individuals.

### 3.3. Echocardiographic Findings

Regarding echocardiographic parameters, patients with long COVID-19 had a lower left ventricular ejection fraction, impaired LV-GLS and greater left ventricular end-diastolic diameter (Table 3). Among other examined echocardiographic parameters, there was no difference between patients and healthy controls in the indices of the right ventricular structure and function, size of the left atrium and indices of increased left ventricular end-diastolic pressure. Overall, 30% of the patients presented with impaired left ventricular performance (LV-GLS > −20%), 12% had impaired right ventricular performance (PASP > 30 mmHg) and 10% presented with both right and left ventricular performance impairment, whilst 48% presented with normal cardiac function.

### 3.4. Associations between Exercise Tolerance and Cardiovascular Function

**Figure 2:**
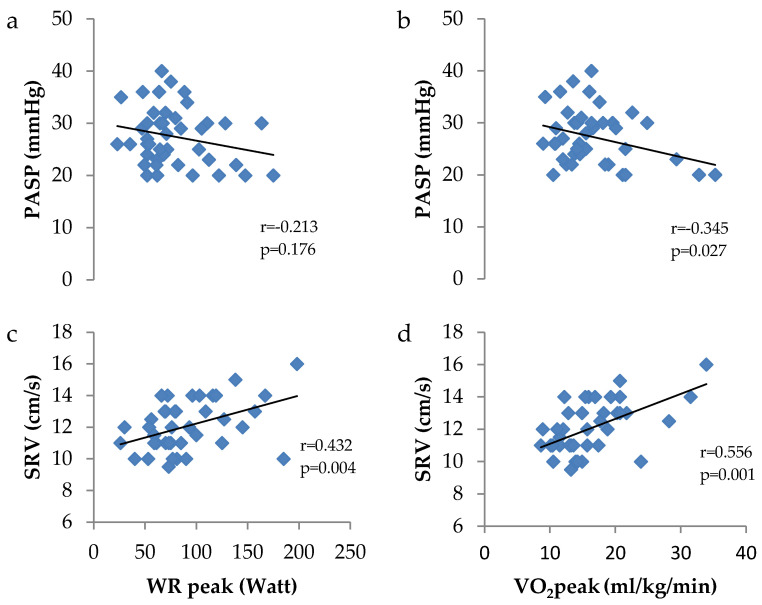
Association between (**a**) pulmonary artery systolic pressure (PASP) and peak work rate (WR), (**b**) PASP and peak oxygen consumption (VO_2_), (**c**) tricuspid annular systolic velocity (SRV) and WR and (**d**) SRV and VO_2_.

**Figure 3:**
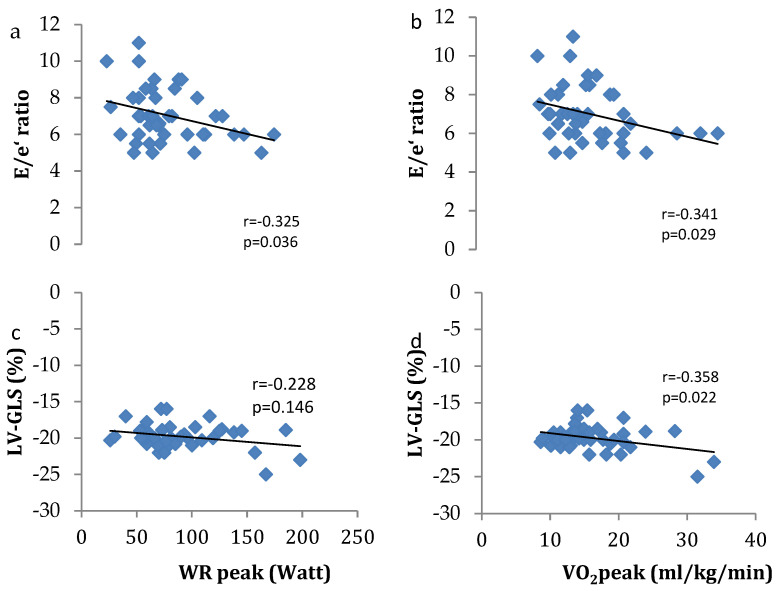
Association between (**a**) trans-mitral flow velocity/early mitral annular velocity (E/e’) and peak work rate (WR), (**b**) E/e’ and peak oxygen consumption (VO_2_), (**c**) left ventricular global longitudinal strain (LV-GLS) and WR and (**d**) LV-GLS and VO_2._

Associations between right heart function and exercise tolerance outcomes are presented in Figure 2. PASP was associated with peak oxygen consumption (VO_2_peak) (r = −0.345; *p* = 0.029), whilst SRV was associated with peak work rate (WRpeak) (r = 0.432; *p* = 0.004) and VO_2_peak (r = 0.556; *p* = 0.001). Associations between left heart function are presented in Figure 3. The E/e’ ratio and LV-GLS were both associated with VO_2_peak (r = −0.341; *p* = 0.029 and r = −0.358; *p* = 0.022, respectively), whilst only the E/e’ ratio was associated with WRpeak (r = −0.325; *p* = 0.036). No significant associations were reported in the healthy controls between right and left heart function and exercise tolerance outcomes.

## 4. Discussion

In this study, we have demonstrated that cardiovascular dysfunction at rest was associated with exercise intolerance in long COVID-19 patients. Specifically, impaired left and right ventricular performance was associated with reduced peak VO_2_ during incremental exercise in patients with long COVID-19 syndrome when compared with age-matched healthy controls.

The magnitude of abnormally low peak VO_2_ in the present study is comparable to that reported in recent studies in COVID-19 survivors at 3, 6 and 11 months post-hospital discharge [20,21,22,23]. However, these studies found no major pulmonary or cardiac limitations in patients with long COVID-19 syndrome, thereby suggesting that cardiac limitations were unlikely to limit exercise tolerance [20,21,22]. Instead, these studies rather suggested that peripheral factors affecting oxygen extraction and utilisation combined with autonomic dysregulation were likely responsible for reduced VO_2_ [20,21,22,23]. In contrast, Szekely and colleagues reported that reduced exercise tolerance was associated with an attenuated heart rate and stroke volume reserve in patients recovering from COVID-19 [24]. Th authors suggested that the inability to increase the stroke volume could result from either left or right ventricular dysfunction [24,25] and diastolic dysfunction [26]. Failure to augment the stroke volume as a potential mechanism of exercise intolerance has also been reported using magnetic resonance-augmented CPET [27] in COVID-19 survivors. Our findings are in line with these studies [24,27], when considering the significant associations between left ventricular function and peak values for WR and VO_2_.

In a recent systematic review and meta-analysis [4], several potential mechanisms were identified to limit cardiopulmonary exercise tolerance in long COVID-19 patients including (i) alterations in systemic oxygen delivery and peripheral muscle oxygen extraction (due to direct myopathic changes from the virus and/or mitochondrial dysfunction) [23], (ii) chronotropic incompetence [26,28] and preload failure [26,29], (iii) dysfunctional breathing [30] and (iv) deconditioning [30]. According to Durstenfeld et al. [4], autonomic and endothelial dysfunction caused by SARS-CoV-2, chronic inflammation and autoimmunity could be responsible for the aforementioned pathophysiologic mechanisms limiting exercise tolerance in long COVID-19 syndrome. Recent evidence has consistently shown that chronotropic incompetence is a key limitation to exercise capacity in patients with heart failure with preserved ejection fraction [31,32]. Similarly, long COVID-19 patients may experience chronotropic insufficiency, which could further impair their exercise tolerance. Our cohort study found that long COVID-19 patients with cardiac dysfunction, albeit without heart failure, exhibited reduced exercise tolerance, which may be attributed to chronotropic incompetence.

In regard to the cardiovascular system, COVID-19 convalescent patients have been reported to present with impaired left and right ventricular systolic performance, impaired endothelial function, increased arterial stiffness and impaired ventriculoarterial coupling [5,6,33,34]. Left ventricular systolic dysfunction is often subclinical without an overt reduction in LVEF, and a number of studies have described the use of cardiac magnetic resonance and LV-GLS in identifying potential subclinical left ventricular dysfunction in long COVID-19 patients [35,36]. Our results are in line with the existing literature as we report that patients with long COVID-19 syndrome presented with impaired left ventricular performance, assessed via LVEDD, LVEF and LV-GLS, compared to healthy individuals, despite that the aforementioned variables were not associated with exercise tolerance [6]. Interestingly, within the three patients with pre-existing coronary artery disease, LV-GLS was only slightly reduced in two patients, and PASP was slightly increased in one patient. As the values in the aforementioned parameters were not outliers and thus did not affect the outcome of the analysis, we decided to include them in the analysis.

The capacity of the cardiovascular system to adapt to increased metabolic requirements, such as those during incremental exercise, cannot be assessed based on a single cardiovascular parameter since the whole cardiovascular performance is based on rather complicated multifactorial mechanisms [37].

LV-GLS has been evaluated as an early index of systolic impairment before any change in the left ventricular ejection fraction is observed [38]. Indeed, investigations have shown that LV-GLS is not only a prognostic indicator but is also associated with the functional performance of the patients [39].

Furthermore, during exercise, oxygen delivered to the tissues should suffice for their metabolic requirements, which is another drive to increase cardiac output. Venous return is also increased during exercise. The required increase in cardiac output is further facilitated if the systolic performance of the right ventricle and its ability to forward blood through the pulmonary circulation are intact [40].

There is evidence of the impact of impaired SRV on exercise tolerance, during the 6 min walking test and cardiopulmonary exercise test [41]. Indeed, in the present study, SRV in patients with long COVID-19 syndrome is correlated with exercise workload. Taken together, these findings strengthen the role of SRV evaluation both at rest and during exercise testing in convalescent COVID-19 patients and may suggest that right ventricular systolic impairment may act as an additional mechanism underlying the poor performance status of long COVID-19 patients.

Furthermore, the adequacy of the left ventricular filling during the diastolic phase of the cardiac cycle determines the left ventricular preload and contributes to enhanced left ventricular performance and hence cardiac output [42]. The increase in heart rate during exercise shortens the diastolic period, and in conjunction with a stiff and non-compliant left ventricle, left ventricular filling is achieved at the cost of higher energy consumption; the subsequent increase in the left atrial pressure is transmitted to the pulmonary circulation causing pulmonary hypertension and/or pulmonary interstitial oedema, thus further compromising respiratory capacity [37]. Left ventricular diastolic impairment and an increased ratio of E/e’—the surrogate index of left ventricular filling pressures—have been reported in post-COVID-19 patients [43,44,45]. Importantly, in the present study, we have reported that an increase in the E/e’ ratio and PASP is correlated with reduced exercise tolerance in long COVID-19 patients.

In our cohort, baseline measurements for FEV_1_, FVC and FEV_1_/FVC were within the normal range in both groups, despite the fact that statistical differences were noted. In fact, neither an obstructive nor restrictive pattern was observed in the patient group. Moreover, SpO_2_ at the limit of tolerance was not different between the two groups even though DLco was mildly reduced in the patient group compared to the healthy individuals. Hence, the reduced DLco is unlikely to account for the differences in exercise tolerance between the two groups.

The ventilatory equivalent for carbon dioxide output (VE/VCO_2_) corresponds to the ventilation required to expire a certain amount of carbon dioxide. The VE/VCO_2_ ratio is defined as pathologic when it is greater than 40 in peak exercise, and this could result from increased dead space in the context of either interstitial lung disease or pulmonary hypertension [46]. In our cohort, the observed differences in the two groups in VE/VCO_2_ were not statistically significant and were within the normal range. In combination with the results from the pulmonary function testing, we deduced that patients did not show signs of interstitial lung disease (normal TLC and mildly reduced DLco). In terms of PASP measurements assessed via resting echocardiography, we found that the values for PASP were slightly increased in both groups, and the differences between the patient group and the controls were not statistically significant.

The healthy group exhibited a more pronounced cardiovascular response to exercise, characterised by an increased heart rate and mean arterial pressure during peak exercise, as compared to the patient group. This enhanced response is likely attributed to the superior exercise capacity demonstrated by the healthy individuals, as reflected by their higher achieved work rates.

### 4.1. Clinical Implications and Future Directions

Our study contributes to the growing body of evidence supporting the notion that the causes of exercise intolerance in long COVID-19 syndrome are complex and multifactorial and that different clusters of symptoms arise from diverse mechanisms [4]. Impaired left and right ventricular performance may reduce exercise capacity in a subgroup of long COVID-19 patients. Regular follow-up and timely interventions by heart and lung specialists are important in addressing possible complications and reassuring patients about the benignity of the condition. Future studies should investigate the effect of cardiopulmonary rehabilitation programmes on cardiovascular function in patients with long COVID-19.

### 4.2. Study Limitations

A few limitations should be taken into consideration while interpreting the results. The population of the control group is relatively too small to provide a practical comparison that would strengthen statistical significance, especially in an older population where there may coexist several physiological factors that limit exercise capacity. Echocardiography was not performed during the CPET, and thus, heart function during exercise could not be assessed in the present study. Additionally, pulmonary artery systolic pressure was estimated via echocardiography to avoid the use of a pulmonary arterial catheter that would have provided a direct measurement. Another limitation was that this was a cross-sectional study, and thus, it was not possible to observe changes over time. Hence, we missed the potential longitudinal effects of COVID-19 on cardiovascular and exercise performance. Finally, data on CPET and echocardiography in recovered COVID-19 patients without long-term symptoms were lacking, preventing us from distinguishing whether the observed cardiovascular dysfunction was a direct result of long COVID-19 or other confounding factors. Consequently, comparisons between survivors with and without long COVID-19 syndrome were not feasible, thereby impeding this study from establishing a clear cause–effect relationship.

## 5. Conclusions

In patients with long COVID-19 syndrome, exercise tolerance is reduced, as evidenced by lower WRpeak and VO_2_peak levels. Additionally, cardiac function is compromised, with impairments noted in both left and right ventricular systolic performance, PASP and diastolic function indices. These cardiac abnormalities are most likely linked to the decreased exercise capacity observed in these patients. It is important to identify the various phenotypes of long COVID-19 and understand the associated underlying pathophysiologic mechanisms. These findings may enable the development and implementation of new therapeutic interventions.

## Figures and Tables

**Table 1:** Patient demographic characteristics.

Variable	Long COVID-19 Patients	Healthy Control	*p* Value
Gender (Male/Female)	19/23	4/6	0.76
Age (years)	55 ± 13	52 ± 8	0.58
Height (cm)	166 ± 11	170 ± 12	0.293
Weight (kg)	80.8 ± 17.8	84.4 ± 20.0	0.579
BMI	29.0 ± 5.2	28.8 ± 3.7	0.916
Days from discharge	149 ± 92	-	-
Smoking status			
Current	5	0	
Ex	6	3	0.57
Never	31	7	
FEV_1_ (% predicted)	98 ± 19	110 ± 17	0.065
FVC (% predicted)	96 ± 21	121 ± 20	0.002
FEV_1_/FVC	84 ± 6	77 ± 6	0.002
TLC (% predicted)	92 ± 27	100 ± 3	0.339
Comorbidities			
Dyslipidaemia	8		
Hypertension	6		
Coronary artery disease	3		
Diabetes mellitus	4		
Asthma	2		
Anxiety–Depression	4		
DLco (% predicted)	73 ± 21	98 ± 3	0.002
mMRC	2.1 ± 1.2	0.4 ± 0.5	0.001
FACIT	24 ± 10	48 ± 4	0.001

DLco, Diffusing Capacity for Carbon Monoxide; FACIT, Functional Assessment of Chronic Illness Therapy scale; FEV_1_, Forced Expiratory Volume at the 1st second; FVC, Forced Vital Capacity; mMRC, modified Medical Research Council Dyspnoea Scale; TLC, Total Lung Capacity; values are mean ± standard deviation (SD).

**Table 2:** Cardiopulmonary exercise testing data at the limit of tolerance.

Variable	Long COVID-19 Patients	Healthy Control	*p* Value
WRpeak (watts)	88 ± 40	148 ± 63	0.001
WRpeak (%pred)	74 ± 27	115 ± 27	0.001
VO_2_peak (mL/kg/min)	16.1 ± 5.7	21.1 ± 4.3	0.014
VO_2_peak (%pred)	67 ± 18	89 ± 13	0.001
RER	1.12 ± 0.10	1.26 ± 0.11	0.001
VE/VCO_2_	34 ± 6	37 ± 6	0.116
O_2_ pulse (mL/kg/beat)	10.4 ± 3.5	11.4 ± 4.0	0.465
O_2_ pulse (%pred)	90 ± 21	95 ± 12	0.495
Heart rate (beats/minute)	126 ± 18	159 ± 17	0.001
Heart rate (%pred)	79 ± 18	95 ± 10	0.007
Mean arterial pressure (mmHg)	108 ± 17	126 ± 14	0.003
SpO_2_ (%)	96 ± 5	99 ± 1	0.078
Dyspnoea	3.8 ± 2.3	4.4 ± 2.8	0.476
Fatigue	5.9 ± 2.5	6.0 ± 2.3	0.890
Reason for termination (Dyspnoea/Fatigue/Both) (%)	4 (10%)/34 (80%)/4 (10%)	4 (40%)/6 (60%)/0 (0%)	-

RER, respiratory exchange ratio; SpO_2_, oxygen saturation; VCO_2_, carbon dioxide output; VE, minute ventilation; VO_2_, oxygen consumption; WR, work rate; values are mean ± SD.

**Table 3:** Resting echocardiographic data.

Variable	Long COVID-19 Patients	Healthy Control	*p* Value
LVEDD (mm)	54 ± 5	48 ± 2	0.001
LVEF (%)	54 ± 5	59 ± 4	0.010
LVmass (g)	135 ± 25	143 ± 29	0.412
LAvol (ml)	45 ± 11	45 ± 10	0.986
E/e’ ratio	7.0 ± 1.5	6.4 ± 1.0	0.230
RV diameter (mm)	31 ± 3	31 ± 4	0.706
SRV (cm/s)	12.0 ± 1.6	12.6 ± 1.8	0.325
PASP (mmHg)	28 ± 6	27 ± 4	0.892
LV-GLS (%)	−20 ± 2	−21 ± 1	0.020
LVOT (mm)	22.8 ± 2.3	22.2 ± 2.4	0.521
SV (ml)	75 ± 9	72 ± 7	0.325

E/e’, early trans-mitral flow velocity/early mitral annular velocity; LAvol, Left atrium volume; LV-GLS, left ventricular global longitudinal strain; LVEDD, left ventricular end-diastolic diameter; LVEF, left ventricular ejection fraction; LVmass, Left ventricle mass; LVOT, Left ventricle outflow tract; PASP, pulmonary artery systolic pressure; RV, right ventricle; SRV, tricuspid annular systolic velocity; SV, stroke volume; values are mean ± SD.

## Data Availability

The data presented in this study are available upon request from the corresponding author.
